# Exploring the synergies between focused ethnography and integrated knowledge translation

**DOI:** 10.1186/s12961-018-0376-z

**Published:** 2018-11-03

**Authors:** Jennifer Baumbusch, Sarah Wu, Sandra B. Lauck, Davina Banner, Tamar O’Shea, Leslie Achtem

**Affiliations:** 1 School of Nursing, University of British Columbia, T201 – 2211 Wesbrook Mall, Vancouver, BC V6T 2B5 Canada; 2 Department of Kinesiology, University of Waterloo, 200 University Avenue West, Waterloo, ON N2L 3G1 Canada; 3 St. Paul’s Hospital, 1081 Burrard Street, Vancouver, BC V6Z1Y6 Canada; 4 School of Nursing, University of Northern British Columbia, 3333 University Way, Prince George, BC V2N4Z9 Canada

**Keywords:** focused ethnography, integrated knowledge translation, knowledge user, health services, transcatheter aortic valve implantation, patient experience, qualitative methodology

## Abstract

**Background:**

Issues with the uptake of research findings in applied health services research remain problematic. Part of this disconnect is attributed to the exclusion of knowledge users at the outset of a study, which often results in the generation of knowledge that is not usable at the point of care. Integrated knowledge translation blended with qualitative methodologies has the potential to address this issue by working alongside knowledge users throughout the research process. Nevertheless, there is currently a paucity of literature about how integrated knowledge translation can be integrated into qualitative methodology; herein, we begin to address this gap in methodology discourse. The purpose of this paper is to describe our experience of conducting a focused ethnography with a collaborative integrated knowledge translation approach, including the synergies and potential sources of discord between integrated knowledge translation and focused ethnography.

**Methods:**

We describe the specific characteristics and synergies that exist when using an integrated knowledge translation approach with focused ethnography, using a research exemplar about the experiences of frail, older adults undergoing a transcatheter aortic valve implantation.

**Results:**

Embedding integrated knowledge translation within focused ethnography resulted in (1) an increased focus on the culture and values of the context under study, (2) a higher level of engagement among researchers, study participants and knowledge users, and (3) a commitment to partnership between researchers and knowledge users as part of a larger programme of research, resulting in a (4) greater emphasis on the importance of reciprocity and trustworthiness in the research process.

**Conclusions:**

Engaging in integrated knowledge translation from the outset of a study ensures that research findings are relevant for application at the point of care. The integration of integrated knowledge translation within focused ethnography allows for real-time uptake of meaningful and emerging findings, the strengthening of collaborative research teams, and opportunities for sustained programmes of research and relationships in the field of health services research. Further exploration of the integration of knowledge translation approaches with qualitative methodologies is recommended.

## Background

Knowledge translation has become a central priority for health researchers, funders, policy-/decision-makers, clinicians and, increasingly, patients and families. This focus in health services research is evidenced by a recent proliferation of knowledge translation research, as well as an increasing focus on patient- and family-oriented research. Yet, knowledge translation can remain problematic despite various dissemination and end-of-grant strategies employed by researchers. Within health services research, this problem is amplified since rapidly changing healthcare systems can render research knowledge irrelevant before it reaches the application stage if left to end-stage knowledge translation strategies alone [1]. This disconnect between research and application, or the “*knowledge to action gap*” [1], can also stem from a failure to produce meaningful and relevant knowledge for users.

To address these challenges, integrated knowledge translation is gaining momentum as a much needed “*involved social process*” [2] to embed in health services research. In this approach, collaborative activities between researchers and knowledge users take place concurrently with the research process, and can include identifying the research questions, selecting the methodology, collecting data, analysing and interpreting findings, and shaping the dissemination approach [3, 4]. Unlike traditional end-of-grant knowledge translation activities, integrated knowledge translation is characterised by a more participatory, non-linear and collaborative approach to relations between researchers and knowledge users, where emphasis is placed on the process as well as on the outcome [4]. Acknowledgement of the socioenvironmental context is embedded within the integrated knowledge translation paradigm, where the “*transcendence of frontiers*” [5] (i.e. sectors, disciplines, geographic location, culture, etc.) and the expansion of integration beyond these frontiers are the guiding principles. Integrated knowledge translation embraces and addresses the complex nature of problems by recognising multi-level interacting factors and diverse sources of evidence [1]. As such, the key ingredient for enhancing the uptake of knowledge is a strong match between patient need, professional consensus, receptiveness of context and leadership facilitation [2]. Unlike more traditional approaches to research that aim for generalisable or transferable study findings, the knowledge generated throughout the integrated knowledge translation research process can be used locally to transform care practices and service delivery as the research study unfolds. The end result of this collaboration between researchers and knowledge users is an efficient and expedient integration of mutually beneficial findings into practice [6] since findings are more likely to be both useful and readily applicable and, importantly, can be used both during and after the research process [1, 7].

To date, little has been written about how qualitative methodologies, such as phenomenology, grounded theory or ethnography, can incorporate an integrated knowledge translation approach. Indeed, integrated knowledge translation is often conflated with participatory action research [8], and yet, while there are elements of integrated knowledge translation that are participatory, it is not participatory action research per se, with the main difference being that integrated knowledge translation looks to bring about change in practice or policy, and participatory action research focuses on social inequalities and oppression [9]. Those studies that have reported combining integrated knowledge translation with qualitative methods do so by leaning heavily on integrated knowledge translation process components, leaving less consideration for the qualitative methodology undertaken [10]. More recently, integrated knowledge translation has been blended within mixed methods, although these designs tended not to employ traditional qualitative methodologies [5, 11–13].

In this paper, we propose focused ethnography as a qualitative methodology in which to embed integrated knowledge translation processes. This methodology grew out of the ethnographic tradition. In the most general of terms, ethnography can be understood as research that “*asks questions about the social and cultural practices of groups of people*” [14], where the priority is placed on gaining an insider’s perspective while still considering the influence of the outsider’s view point [15, 16]. Ethnographies attempt to capture truthful accounts of people’s experiences using their own words, where the importance of context is accounted for as the researcher is immersed in the social world of participants [17]. While inherently staying true to the roots of ethnography as a way of describing a culture by learning about people from them [17], focused ethnography examines a specific topic or subculture, explicitly linking the micro and macro, and making it well suited to practice-based research such as applied health services [17, 18].

More recently, there has been a shift in health research towards gaining a better understanding of patient perspectives to augment quantitative studies that tend to focus on clinical outcomes; focused ethnography is a methodology to promote this shift [19, 20]. Examples of studies that have employed focused ethnography include research about the role of the physical environment on older adult care in the Emergency Department [21], the experience and perceptions of community mental health nurses delivering therapy sessions [22], and the relationship between perceptions of the risk of falling of older adults and their adult children [23], to name a few.

In this paper, we begin to address a gap in methodological scholarship and reflect upon our experiences of conducting integrated knowledge translation in concert with the qualitative methodology of focused ethnography. We first situate the paper in an exemplar from our research programme, which examines frail, older adults’ experiences of care processes in a transcatheter heart valve programme, a health service for people with advanced heart disease. We then continue by describing the characteristics of focused ethnography and of an integrated knowledge translation approach, followed by the synergies that exist between the two. We conclude by discussing the lessons learned, including the strengths and limitations of conducting integrated knowledge translation in concert with focused ethnography.

## Methods

### Exemplar: transcatheter aortic valve implantation (TAVI) study

#### Aim

The study that provides the context for the current exploration of the synergies between focused ethnography and integrated knowledge translation forms part of a larger research programme exploring frail, older adults’ and their family caregivers’ experiences of undergoing TAVI, which is an innovative, minimally invasive heart valve procedure. Our research complements ongoing clinical trials and ensures that patient experiences are reflected in developing and refining care processes. The purpose of the study presented here was to examine patient and family caregiver journeys from the point of eligibility assessment to post-procedure recovery at home.

A focused ethnography methodology was employed in this study because it (1) allows for intensive, in-depth qualitative data collection over a short period of time, and (2) emphasises the generation of knowledge that can be translated into practice-ready strategies in real time. Because of the rapidly changing nature of care processes associated with TAVI, it was important to use a methodology that could be responsive and flexible to the changes in care delivery and also offer insights to help inform these developing and evolving care processes. Over the course of the study, for example, the recommended post-procedure hospital stay decreased in length [24]. During the post-procedure interviews with participants, we were able to learn about patients’ and caregivers’ experiences of this change and share this information with clinicians.

#### Setting and participants

The study took place at a provincially coordinated cardiac centre located in Western Canada, which has been a pioneer in developing transcatheter heart valve procedures. Patients who are referred for TAVI have multiple points of contact with the procedure team at the provincially coordinated site, while receiving ongoing care from their community-based providers. Patients initially undergo an assessment for eligibility, which requires a visit to the procedure site. If eligible, they return for a pre-admission clinic visit, and are then admitted for the procedure. Patients living in closer proximity to the procedure site have three on-site assessments, and those living at a distance have two on-site visits (pre-admission and procedure are typically combined for these patients to minimise travel requirements).

To recruit participants, we mailed study invitations to patients referred for TAVI and then followed up with a phone call. Inclusion criteria to participate were (1) ability to converse in English and (2) age 65 years and older. Purposive sampling was employed to explore patient differences; in particular, patients were identified as ‘in town’ (living within 100 km radius to procedure site) or ‘out of town’ (living beyond 100 km radius of the procedure site and/or required to cross the sea when travelling to the procedure site). We also recruited around availability of informal supports and patient participant gender. While other patient and caregiver characteristics, such as ethno-cultural background, would contribute to the experience of undergoing the TAVI procedure, this study only includes those who were able to communicate in English. Diversity in ethno-cultural background was not explicitly sought out in this study and we recognise that this is a limitation. However, it is important to note that currently there is no evidence that identifies a higher proportion of specific ethno-cultural groups that may be more prone to cardiac issues, such as atrial fibrillation, that would result in a TAVI referral. In total, 31 patients and 14 family caregivers (i.e. spouses, adult children, friends) (*n* = 45) participated in the study. Following their initial assessment, 18 of the 31 patient participants were eligible to undergo the TAVI procedure.

#### Data collection and analysis processes

Data collection entailed semi-structured interviews and participant observations and took place throughout the patient care journey. Up to three interviews were conducted per patient participant, namely (1) at the time of referral, (2) within 1 week of the procedure, and (3) at 1-month post-procedure. During the process of obtaining informed consent at each point of contact, participants were informed that anonymised aggregated data would be shared with clinicians at the study site in order to immediately begin the process of informing and refining care processes. During the interviews, participants were specifically asked about recommendations to improve care processes. Participant observations took place during the eligibility assessment clinic visit and during the in-patient stay post-procedure. The time leading up to and following cardiac procedures are often highly stressful for both patients and informal caregivers. In order to ensure that study participants felt supported through the data collection process, expert TAVI clinicians who were part of the research team (i.e. co-authors SL and LA) were available for follow-up for any issues and/or concerns that arose during the study. As is part of the research ethics process in this study, all research materials provided to participants during the consent process emphasised the availability of expert clinicians for information and support; this was reiterated by the research trainees at the beginning of every data collection point. Further, as research trainees were selected for the study to avoid bias that otherwise would have been present if more experienced staff or clinicians had been involved in data collection, regular debriefing sessions were held with the research team in order to process any difficult issues that arose during the data collection process. Over the course of the study, we conducted a total of 74 interviews and 33 participant observations. Data analysis occurred concurrently with data collection. Issues and trends related to care processes that were identified in the early stages of analysis were shared with clinicians throughout the research process.

#### Integrated knowledge translation processes

Importantly, the programme of research in which this study was situated is grounded in a long-standing research collaboration between academic-based researchers, clinical scientists embedded in the healthcare system, and point-of-care clinicians from medicine and nursing. In this study, those who were in a position to effect TAVI care processes, for example, those who were involved in both the day-to-day clinical care of TAVI patients and also those involved in developing provincial policy, were identified as the knowledge users. It is for this reason that patient participants themselves were not included on this research team; however, due to the heavily involved nature of focused ethnography, patients were direct beneficiaries of those findings, as some were immediately embedded within the TAVI clinic’s care processes. While this study primarily focused on building researcher–clinician relationships, subsequent studies among this research team have increasingly involved patients across the research process. Table 1 provides a clear outline as to how key terms were defined in this study.

**Table 1:** Key terminology and definitions

Term	Definition
Focused ethnography	A type of ethnography whereby a specific topic or subculture is investigated using multiple types of data collection methods [25]
Knowledge translation	An iterative process that involves synthesising, disseminating and exchanging knowledge with the intention of improving health delivery systems and the health of a population [40]
Integrated knowledge translation	A collaborative research venture undertaken by researchers and knowledge users with the intention of generating knowledge that is meaningful and mutually beneficial [1]
Research team	Those who are responsible for carrying out the study protocol; members of the research team included one academic researcher (Principal Investigator), one clinical scientist (Co-Investigator), one academic researcher (Co-Investigator) and six research trainees
Knowledge user	Those who have direct influence over the policy and procedures of the health services; this group was involved in identifying research gaps, formulating research questions, informing research methods, and disseminating and enacting findings [1] Knowledge users included one TAVI programme coordinator, one TAVI programme manager, two interventional cardiologists and one clinical scientist (Co-Investigator) who was the primary liaison between the research team and the knowledge users
Patient participant	Those who are engaged in the process of undergoing the TAVI procedure; 31 patients participated in the study
Family caregiver	Those identified as the main source of tangible, emotional and/or informational support for the patient undergoing the TAVI procedure; 14 family caregivers participated in the study

Regular study status updates were circulated to team members in order to provide information about the progress of data collection and analysis. Teleconferencing, emails, and face-to-face meetings were used to facilitate ongoing communication and feedback. While a core group of team members focused on the more academic-oriented research processes, these communications with the larger team allowed for real-time uptake of emerging findings at a very practical level.

An example of a practical strategy was the creation of a fact sheet for patients traveling to the procedure site. Through data collection we learned that patients living more than 100 km from the procedure site were often incurring significant out-of-pocket expenses. The research team and clinicians created a fact sheet that included information on ways to save and/or submit reimbursements for travel expenses, and provided a map of affordable hotels that were close to the procedure site. Many of the suggestions were from patients and family caregivers who had experienced traveling to the procedure site.

## Results

In this section, we begin by describing focused ethnography and integrated knowledge translation as they were implemented in our study. We then explore the synergies between the two approaches and describe how these were enacted in the study.

### Accounting for context in focused ethnography

There are several characteristics that are distinct and essential to focused ethnography, as identified by Knoblauch [25]; while some of these characteristics are unique and others are similar to traditional ethnography, taken together, they create the novel methodology of focused ethnography. The first characteristic identified is ‘short-term field visits’, as opposed to long-term immersion, most often in the form of short, intense, non-continuous intervals. Duration in field is the most distinctive differentiation between traditional and focused ethnography. As such, a critique is that data gathered during this shorter time period is ‘superficial’. However, intense data collection using various types of data (for example, audio and video recordings) counterbalances compressed field time [25]. Our study involved intense data collection sessions over a duration of several months for each participant. Patient participants who were ineligible for the TAVI procedure had two data collection points and those who were eligible had up to six data collection points. Additionally, field time was dependent upon the nature of the context and activity observed. For example, pre-assessment clinical observations typically lasted, on average, 30 minutes, whereas telephone interviews lasted anywhere between 45 and 90 minutes.

A second characteristic is the generation of a large amount of data. In our study, we conducted a total of 74 semi-structured interviews with patients and family caregivers. We also conducted 33 participant observations, which included conversations among patients, their family caregivers and clinicians. Observations were conducted with the intention of capturing what was required of patients and their family caregivers within this context, to experience events and their significance in ways that allowed the observer to approximate participants’ experiences [26]. In addition to these ‘formal’ aspects of data collection, analytical memos were written as needed and documents, such as patient education materials, were reviewed.

A third characteristic is intensive data collection, whereby various recording devices are considered equivalent to human observation techniques [25]. During the study, data were collected in the form of field notes based on participant observations (i.e. clinic assessments), memos, telephone interviews (transcribed), face-to-face interviews (transcribed), and document analysis (i.e. medical chart audit). Instances where data were technically recorded, for example, during transcribed telephone interviews, allowed select expert groups, which had varying knowledge backgrounds, the opportunity to interpret and analyse findings. Further to this, Knoblauch [25] argues that technically recording data also permits the observer time to focus on specific features of a group or inquire further about an event, as opposed to attention spent making hand-written recordings. In this way, traditional, objective participant observation is not the aim and instead is replaced with an engaged ‘field-observer’ role [25]. Implications of recording data using technical devices, such as recorders, also means that the observer is free to make observations, ask questions and reflect, moving the ethnographer closer to the emic perspective [25]. Throughout the TAVI study, research trainees (e.g. undergraduate and graduate students) captured interviews using a digital recorder, allowing them the time to concentrate on inquiring and prompting in a more personalised manner, while building rapport with participants. Given the highly stressful context under which these older adults were participating (i.e. waiting for or receiving a valve replacement), building trust in relationships with participants was of utmost importance. Interviews in which fellowships were formed between research trainees and participants were longer in length, with richer and more insightful reflections from participants. As patients progressed in their TAVI journey, participants were noted as expressing eagerness and enthusiasm to speak with the research trainees; these feelings were mutual for the research team. This reciprocal commitment was reflected in the number of those patients who were still willing to participate for their final interview; of the patients who received the TAVI procedure (*n* = 18), 12 carried out a fourth interview several months after initial recruitment. Important to mention is the special attention required to maintain informed consent from participants. As there were multiple data collection encounters with several different research trainees over the span of several months, on-going consent was exercised throughout the study at each data collection time point.

A fourth characteristic is collective data analysis, whereby data collected by multiple individuals are collectively analysed and interpreted as a team [25]. In this way, analysis occurs in groups, ideally comprised of members with sufficient diversity in social and cultural background, and yet still possess adequate background knowledge of the field in focus [25]. By analysing data as a group, there was a deeper understanding and appreciation of findings, as knowledge users could provide cultural insights (insider view) and members of the research team could provide an external interpretation of data (outsider view). In this way, these two groups offered heightened perspectives to the analysis process that otherwise would not have been identified had analyses been performed individually by the research team [17]. Knowledge users, therefore, have an integral role in shaping the research process from the time of developing research questions through data analysis and interpretation. Throughout our study, clinical research team members from the study site were provided with weekly data collection updates by the research trainees. Once preliminary analysis began, regular meetings were held, where group members were provided with cleaned raw data for review and collective data analysis began. Present were the Principal (i.e. academic researcher (JB)) and Co-Investigators (i.e. clinical scientist (SBL), TAVI programme coordinator (LA), TAVI programme members, other academic researchers, research trainees) (Table 1).

Discussions during these meetings revolved around emerging findings, areas that warranted more attention during data collection, and a review of interview and observation guidelines and other research process issues. The dialogue that arose from these group sessions re-focused data collection strategies, identified solutions to data collection problems that periodically arose, and assisted in the development of an analysis codebook. Much like the iterative nature of data analysis, these group sessions created opportunities for knowledge users and researchers to continually revisit and hone in on critical aspects of the field in focus. It was through these dialogue sessions that, for example, the idea of a fourth interview was generated in order to gain greater insight into the recovery period at home, which could then inform pre-admission patient teaching. Moreover, this process provided real-time feedback (i.e. personal accounts from interviews and observations) from patients who otherwise would not have had opportunities to relay their unique experiences in such detail. Further, regular meetings with researchers and knowledge users allowed for the establishment of a safe and open environment, where the realities of patient experiences moving through the TAVI process could be candidly discussed. Group data analysis was recorded by a research trainee and included changes to the data collection protocol and changes in the working definitions of concepts and themes as analyses progressed, as well as rationales for changes in the form of memos.

In sum, focused ethnography places great emphasis on the value of *“everyday actions or inaction*” [16] and the broader culture or context of healthcare services, lending itself to a movement towards more effective and appropriate uptake of research knowledge for everyday use. In particular, the scope and intensity of focused ethnography makes this methodology particularly effective when applied in conjunction with an integrated knowledge translation approach, which is described in more detail in the following section.

### Fostering partnership through integrated knowledge translation

The application of integrated knowledge translation is most appropriate in situations where the ‘problem’ is identifiable [27]. In some cases, the motivation for the research may have stemmed from a knowledge user who felt compelled to seek assistance from academic sources [27]. For example, the genesis of our research partnership between researchers and knowledge users was a point-of-care research study investigating patients’ decisions to undergo eligibility assessment for the TAVI procedure [28]. The Nurse Clinician (co-author LA) who led this study identified an aspect of the TAVI programme that required further exploration. Additionally, there was a recognised need to complement ongoing clinical trials with qualitative research that focused on patient and family caregiver perspectives and experiences on care processes and quality of life following TAVI.

A key distinction between integrated knowledge translation and more traditional knowledge translation is a shift away from linear and unidirectional transfer of knowledge from researcher to user to a more fluid and multi-directional approach [1]. Reciprocity between researchers and users is central to integrated knowledge translation, where researchers bring a distinct set of skills and resources, and knowledge users possess expertise specific to the issue being studied [27]. Elements of integrated knowledge translation have been present in several research disciplines, including collaborative research and participatory action research [3, 7]. As this approach continues to prove itself an essential component of the research process, it is important to acknowledge integrated knowledge translation’s distinctive qualities, namely that researchers and knowledge users must (1) share in the development of the research questions, (2) collaborate on the interpretation of study findings, and (3) collaborate in the delivery of results so that the movement of research findings to practice is meaningful and deliberate [27].

Although these features are applicable to a variety of research fields, they are particularly suited to the rapidly evolving nature of applied health services research. Most often criticised for its lengthy pause between the completion of research and its adoption into practice, research in the field of health services is in need of addressing the issue of knowledge production, namely that research fails to address the most pertinent questions posed by point-of-care staff, management and policy-makers at the outset [1, 29]. Movement away from a traditional biomedical mindset in conjunction with the removal of interdisciplinary barriers towards efforts that embrace healthcare knowledge as both a social construct and complex social processes has the potential to engage and create knowledge within the healthcare organisation at individual, group and corporate-wide levels [1, 5]. Through the identification of a social need and a heavy involvement of knowledge users, integrated knowledge translation provides a level of expertise that allows access to stakeholders and a deeper understanding of context and environment that gives way to faster and more effective uptake of research findings [27]. Our research programme, which has grown from the original point-of-care research study mentioned above, has expanded to include clinician, operational leader and policy-maker knowledge users, in addition to the academically situated researchers.

### Synergies between focused ethnography and integrated knowledge translation: towards reciprocity

**Fig. 1:**
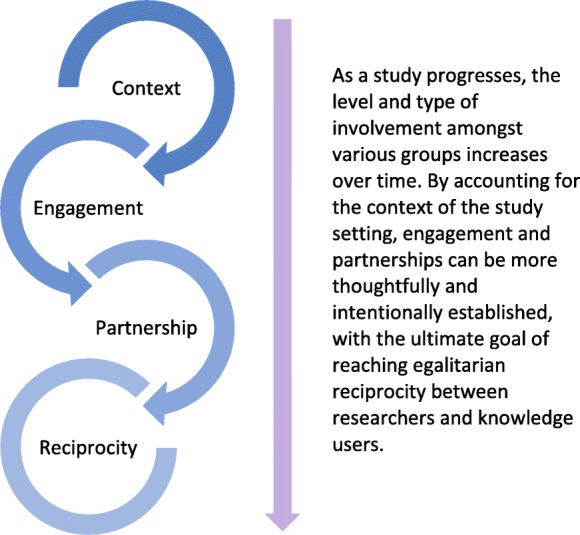
Increasing level of involvement between researchers and knowledge users when embedding integrated knowledge translation within focused ethnography

Thus far, we have described how elements of focused ethnography and integrated knowledge translation were enacted in our study. Bowen and Graham [1] identify the poor uptake of research findings as a direct result of insufficient attempts at addressing identified issues by oversimplifying them as clear-cut cause and effect scenarios, when instead they are a complex interplay between individual players and their larger context. As discussed earlier, the integrated knowledge translation approach places great emphasis on understanding context and the potential for integration across frontiers – aspects also reflected in focused ethnography and the starting point for establishing a basis for mutually beneficial relationships between researchers and knowledge users. Therefore, the components of focused ethnography have the ability to attend to issues surrounding knowledge production by gaining a holistic account of the complexity surrounding a phenomenon. As this methodology is inherently iterative, it allows for opportunities to integrate components, such as integrated knowledge translation, into the process [1]. In this section, we consider the synergies between focused ethnography and integrated knowledge translation (Table 2), making note of the temporality of these complimentary characteristics, whereby one builds upon the other in an effort to move towards the goal of forming a reciprocal relationship between researcher and knowledge user (Fig. 1).

**Table 2:** Synergistic characteristics of focused ethnography and integrated knowledge translation

Focused Ethnography	Synergies	Integrated Knowledge Translation
Purposive sampling to target those with contextual knowledge and experience	← CONTEXT →	Ongoing collaboration between researchers and knowledge users to produce knowledge relevant to context
High level of engagement during data collection and analyses so that knowledge generated is applicable and accessible to knowledge user	← ENGAGEMENT →	Building relationships with knowledge users from the outset helps to identify gaps in knowledge and services through collective interpretation and contextualisation of research findings
Iterative approach allows for flexibility in supporting knowledge user involvement throughout study process, which helps to establish scientific rigour of the study findings	← PARTNERSHIP →	Hierarchies are flattened between knowledge users and researchers so that most relevant research priorities can be identified
Heavy reliance on participant observations requires careful navigation of intersubjective experiences of the researcher and participant	← RECIPROCITY →	Mutually beneficial relationships when interpreting and using study data will help to ensure that relationships formed outlast the project itself

First, inherent to both focused ethnography and integrated knowledge translation, is the need to consider and examine the culture and values of a specific context [2, 5, 17], which entails engaging with those closest and most familiar with the area of inquiry. In focused ethnography, for example, we employed purposive sampling, which targets participants with a distinct set of knowledge and experiences that can inform the study’s investigation [16]. Dedicated time spent establishing relationships with these participants is also part of focused ethnography, and allows researchers to effectively place themselves in a position to contextualise a phenomenon within its socio-political realm [10]. Similarly, intrinsic to integrated knowledge translation is the production of knowledge relevant to users – this is generated in instances where knowledge users feel an affinity towards findings. For our research team to gain a rich understanding (both explicit and tacit aspects) of the patients’ experiences throughout the TAVI programme, the researchers simultaneously engaged with patients, their family caregivers and the clinical team in formal (regular meetings, weekly study updates, interviews, participant observations) and informal (conversations during data collection) situations, as is outlined in Table 3. This engagement also activated an ongoing examination of the power dynamics among researchers, clinicians, and patients and family caregivers. Difficult or uncomfortable contextual factors that surfaced through this focused ethnography, such as inequities in access with some patients having out-of-pocket expenses to access the procedure site, became more palatable to the knowledge users because they were actively involved in identifying those tensions [2].

**Table 3:** Focused ethnography (FE) and integrated knowledge translation (IKT) synergies enacted

IKT and FE Synergy	Component	Point in Research Process	Groups Involved	Enactment
Accounting for context	1. Purposive sampling of participants	Immediately before and during data collection	RT, KUs	Purposive sampling strategy requires the RT and KUs to work closely together at the outset of the study in order to suffice inclusion/exclusion criteria, as well as to identify those who have unique knowledge and experience relevant to the study’s investigation RT and KUs communicated via weekly study update emails (i.e. recruitment status and data collected), bi-monthly in-person clinic meetings to identify new TAVI PPs for purposive sampling, and semi-annual in-person meetings held at research institute to strategise data collection based on aggregated PP characteristics
	2. Simultaneous relationship building	Throughout data collection and analyses	RT, KUs, PPs, FCGs	Simultaneous relationship building between the RT with KUs, PPs and FCGs allowed for in-depth understanding of multiple perspectives within this setting RT gained new insights about the complexity and intersectionality within this context by concurrently engaging with PPs and FCGs (observations and interviews over the course of PP treatment), and positioning these experiences against the health service realities faced by KUs Bi-monthly in-person clinic meetings, data analyses and semi-annual in-person meetings between RT and KUs permitted the time and collective agreement about arising issues that were then contextualised
High level of engagement	3. Consistent and frequent contact with various groups	Prior to participant recruitment and throughout data collection, analyses, and dissemination and uptake offindings	RT, KUs, PPs, FCGs	Consistent level of communication throughout the study is required in order to form meaningful working partnerships, which then shape the applicability and acceptability of research findings RT were in weekly contact with KUs in order for decisions to be made collectively around PP and FCG recruitment, processes around data collection by the RT, data analyses, and uptake of key findings into clinic processes meant to support PPs and FCGs in their experiences Similarly, the RT provided additional informational and emotional support to those PPs and FCGs who found the TAVI experience particularly challenging (i.e. additional time to discuss concerns during interviews, development PP informational pamphlet) Regular meetings meant that these issues were acknowledged and accounted for immediately
Establishing partnerships	4. Effort to flatten knowledge hierarchy	Power dynamics acknowledged and addressed prior to and throughout study processes	RT, KUs	RT and KUs worked to relax restrictive distinctions between researchers and clinicians through continuous engagement in order to establish and maintain a productive partnership KUs identified gaps in literature and practice to help form relevant research questions Precedent was given to KUs around decisions pertaining to on-going study processes during weekly and bi-weekly meetings All data generated was made accessible to KUs (once cleaned for personal identifiers) Mentorship of KUs to RT (i.e. research trainees) through sharing of specialised knowledge and skills relevant to clinical settings during data collection
Striving for reciprocity	5. Acknowledging give and take during the research process	Throughout study processes and beyond conclusion of study	RT, KUs, PPs, FCGs	Shared decision-making around all aspects of the research process Continuing to develop further studies and a programme of research based on partnership between researchers and KUs

*RT* research team, *KU* knowledge user, *PP* patient participant, *FCG* family caregiver

Second, the overall high level of engagement among researchers, participants and knowledge users is another shared feature of integrated knowledge translation initiatives and focused ethnography*.* The establishment of mutually beneficial relationships – built upon an understanding of context – are essential in ultimately shaping applicability and accessibility of the knowledge generated; this cannot be achieved without a consistent level of communication and collaboration throughout the study between the research team and knowledge users. As is with focused ethnography, discussions around data collection processes, concurrent data analyses and the identification of emerging findings, for example, required weekly communication and input from both researchers and knowledge users in order for ideas, opinions, and issues to be heard and acknowledged. Consistent and considerable time was required in order for ideas and problems to be thoroughly addressed as a collective. A similar level of engagement is also needed between the researcher and the study participant when using integrated knowledge translation that requires additional time and energy from both parties. In the case of the study exemplar, some patient participants felt particularly vulnerable due to severe health issues while waiting for/recovering from the cardiac procedure. The research team made every effort to provide emotional and practical support to participants as they each made their transition through the TAVI process, for example, providing directions from the hotel to the hospital for those arriving from out of town, or providing reassurance when discussing fears of dying if something were to happen during the procedure. Time spent understanding participants’ experiences and relaying these immediate and intense feelings from participants to the researchers and knowledge users on the team allowed clinicians to respond accordingly, all the while working towards building rapport and trust with study participants.

Third, focused ethnography and integrated knowledge translation are complimentary in that they view the researcher and knowledge user as partners, permitting synergistic iterations that will result in more relevant and practical applications of findings [3]. The iterative approach that is taken when conducting focused ethnography allows for flexibility in supporting the involvement of multiple key informants and varying sources of knowledge and foreknowledge throughout the study process. Integrated knowledge translation also requires a commitment that precedence be given to concerns identified by knowledge users, where conscious efforts are made to flatten the knowledge hierarchy through the relaxation of restrictive distinctions between researchers and clinicians, knowers and non-knowers [2]. As mentioned earlier, this process of forming partnerships begins at the outset of a research study, yet it cannot be fully realised without an in-depth appreciation for the influence of the study context, as well as consistent contact and engagement between researchers and knowledge users. The study team worked with knowledge users to identify gaps within the literature, practice and policy that affected the delivery and receipt of care of their specific patient population, which then led to the development of relevant research questions and framing of the study. Knowledge users played critical roles during the data collection and analysis phases, where they regularly met with the researchers to reflect on data collection strategies and check, confirm, and reject findings that emerged from the data. Knowledge users also provided on-site support to data collectors, and providing mentorship and training in the clinical setting. Qualitative research relies on several methods to establish rigour or “*trustworthiness*” (e.g. credibility, reflexivity, reciprocity, voice, praxis) [30], each one engaging the researcher in a dialogue about their relationship with participants and context, to ensure that findings are accurately portrayed and honour participants’ voices and realities [31].

Promoting equal partnerships and establishing continuous dialogue between the researcher and knowledge user gives way to a fourth synergy – reciprocity – essential in undertakings of a participatory nature (i.e. integrated knowledge translation and focused ethnography) and an exercise of trustworthiness. Put simply, reciprocity is an “*exchange between social equals*” [32], with the expectation of return – a moral weight – that is only relieved when the exchange has been met [33]. Given the highly hierarchical nature of both the healthcare system and academic institutions, working towards “*egalitarian reciprocity*” [34] is a complex endeavour and requires active reflexivity about the dynamics of relationships and negotiations of power through the understanding of context, time spent in consistent engagement and the formation of productive partnerships. It is difficult, sometimes, to articulate what elements are involved in reciprocity and how one concerns oneself with what should be given and received, and by whom [35]. Harrison et al. [35] offer rapport, safety, honouring and obligation as issues included in the give-and-take of this research approach. When combining focused ethnography and integrated knowledge translation, the “*dualism*” Doane et al. [2] refer to when discussing ‘knowledge’ versus ‘practice’ expands to include the ‘experience’ (patients) of those affected by ‘knowledge’ (researchers) and ‘*practice*’ (clinicians). Because focused ethnography is heavily reliant on participant observations, the intersubjective experiences of patient–researcher–knowledge user created in integrated knowledge translation initiatives requires careful consideration when interpreting and using data in order to honour this joint creation of knowledge and insight. Reciprocity in this context, then, entails the building of a collective perspective based on a mutually beneficial process, with the intention that relationships formed will outlast the project itself [3, 4]. For example, there were several patient participants and their families (*n* = 7) who continued their relationship with the researchers and knowledge users by participating in a 1-year follow-up TAVI study. In addition, academics and clinicians worked towards egalitarian reciprocity, as such being sensitive to ‘home soil’ and ensuring that in-person meetings alternated between hospital and university settings. Moreover, the research team worked to engage and honour each other’s perspectives and ideas through practical acts such as clinicians requesting the addition of a new data collection tool or academics offering suggestions to further modify an interview guide. In essence, it is through previous synergistic components (as detailed in Table 3) that such groups engaged in this collaborative research model may move closer towards reaching egalitarian reciprocity.

## Discussion

By intentionally weaving integrated knowledge translation within a focused ethnography, we have identified several synergies that could help advance the uptake of knowledge in applied health services research. Through this process, we have identified particular strengths as well as challenges in this approach, which are discussed later in this section. At a time where evidence-informed practice and standardisation of care is emphasised, blending qualitative methods with integrated knowledge translation allows for the integration of the individual patient’s perspective in care processes. Additionally, with an increasing focus on patient and family engagement [36], this approach to research ensures the integration of insights from patients and families in the actual generation of new knowledge that can form the foundation for the creation of health service delivery. While patients and their family caregivers were not formal knowledge users in this context, they were certainly considered key stakeholders, and their feedback was considered critical to the production of relevant and meaningful findings.

Our experiences add novel insights into both focused ethnography and integrated knowledge translation. First, little attention has been given to the unique aspects of rigour for focused ethnography methodology, which has often been considered comparable to other types of ethnography [16, 25]. In our research programme it is clear that reciprocity is an important aspect of rigour, not only in focused ethnography but also in integrated knowledge translation, where the triangulation of perspectives makes research findings more meaningful and applicable [37]. Second, our experiences offer an innovative and feasible way in which researchers can embed integrated knowledge translation initiatives into qualitative research designs. As knowledge translation efforts have long emphasised end-of-grant-oriented initiatives, health services researchers and knowledge translation experts are seeking ways in which to incorporate integrated knowledge translation components into traditional research designs. This recognises that these blended models are distinct from other collaborative research approaches, while being best suited to answer the research question [38]. In this way, our study exemplar offers a unique typology of integrated knowledge translation models that can be carried out in health services research, and helps to fill a theoretical gap that exists around this emerging research approach [10, 38].

Intentionally embedding integrated knowledge translation in fluid qualitative methodologies like focused ethnography has several strengths. The intensive data collection of focused ethnography, along with concurrent data analysis, allows for real-time application in care processes. In terms of relationships, the high level of engagement of knowledge users in this approach means that they are invested in applying the study outcomes. The close relationships between academia and practice in this research programme have led to a series of funded studies to examine the perspectives of patients and family caregivers. We acknowledge that a long-term relationship between clinicians and researchers was foundational to the success of this study. As described in Baumbusch et al. [7], a collaborative approach to integrated knowledge translation requires a high level of commitment from both researchers and clinicians and that there are often ‘champions’ in each group who are central to this approach. In our situation, this study was a secondary study within a research programme, and therefore the relationships were already established. We were also fortunate that there was no turnover of clinicians during the course of the study. To sustain these relationships, researchers and clinicians need to regularly meet even during times when there are no active studies in order to discuss potential research questions and consider the next steps in the research programme. Such relationships do not have to be high cost, they rely on less formal, but critical, ongoing activities (e.g. going for coffee, ‘checking in’ regularly by email). For our team, integrated knowledge translation is not limited to a single study, but is a sustained partnership over a series of studies that build upon each other.

In terms of qualitative methodologies, there is opportunity for research teams to further develop integrated knowledge translation models, which are needed in the implementation science literature [10]. Moreover, as focused ethnographies work to link the micro to the macro, they accompany the evolution of integrated knowledge translation towards its third generation, which takes a “*system-level approach*” with the intention to accommodate non-linear features of healthcare [2].

There are also potential limitations and challenges in this approach. In our experience, integrated knowledge translation is highly relational, and therefore consistency in research staffing is important. Over the course of the study there were six research trainees who assisted with data collection and had contact with study participants, knowledge users and researchers. While each brought a valued perspective to the study, they also required training to manage the intensive data collection in a complex clinical setting, and establish relationships with study participants and knowledge users. Trainee turnover also highlights the need for researchers to be highly engaged and in regular contact with knowledge users and to not rely on research staff to maintain these relationships. While trainee turnover was somewhat disruptive to the research process itself, turnover did not impact study participants as trainees were asked to ‘follow through’ with those participants with whom they had first contacted. Because recruitment took place over the course of a year, it was possible for newer trainees to connect with new participants, thus avoiding the issue of participants having to interact with several different trainees. There are also additional requirements when integrated knowledge translation is embedded in an ongoing study such as research staff time to form partnerships with knowledge users, engaging in on-going knowledge translation activities, and travel for face-to-face meetings. When applying for grants, these activities must be considered as central to the success of the research in order to obtain funding [39]. Knowledge users also needed to balance their involvement in the research process with their regular clinical responsibilities. While we tried to organise meetings around their convenience, in the busy context of healthcare, it was difficult and demanding at times for them to maintain a high level of engagement. Lastly, in the study described in this paper, we did not include patients and family caregivers are core knowledge user team members. With increasing focus on patient- and family-oriented research, their presence should be integral on our team.

## Conclusions

In conclusion, qualitative methodologies, such as focused ethnography, can and should be conducted in concert with integrated knowledge translation. The development of innovative methodologies that bridge traditional qualitative approaches with integrated knowledge translation are needed to address current healthcare challenges and ensure the continued relevance of qualitative research in the healthcare sector. Moreover, in a rapidly changing context of health services, a dual approach can be responsive to examining health innovations and translating emerging knowledge in real time to support evidence-informed care. The shared language created by the synergies between focused ethnography and integrated knowledge translation allowed our team, comprised of researchers and knowledge users, to pursue a common aim of improving care processes for frail, older adults.

## Abbreviations

TAVI: transcatheter aortic valve implantation
